# Applications of *q*-Borel distribution series involving *q*-Gegenbauer polynomials to subclasses of bi-univalent functions

**DOI:** 10.1016/j.heliyon.2024.e34187

**Published:** 2024-07-09

**Authors:** T. Al-Hawary, A. Alsoboh, A. Amourah, O. Ogilat, I. Harny, M. Darus

**Affiliations:** a Department of Applied Science, Ajloun College, Al-Balqa Applied University, Ajloun 26816, Jordan; b Department of Mathematics, Philadelphia University, 19392 Amman, Jordan; c Mathematics Education Program, Faculty of Education and Arts, Sohar University, Sohar 3111, Oman; d Jadara Research Center, Jadara University, Irbid 21110, Jordan; e Applied Science Research Center, Applied Science Private University, Amman, Jordan; f Department of Basic Sciences, Faculty of Arts and Science, Al-Ahliyya Amman University, Amman 19328, Jordan; g Department General Education, Faculty of Resilience, Rabdan Academy, Abu Dhabi, United Arab Emirates; h Department of Mathematical Sciences, Faculty of Science and Technology, Universiti Kebangsaan Malaysia, 43600 Bangi, Selangor, Malaysia

**Keywords:** 30C45, *q*-Borel distribution series, Subordination, *q*-Gegenbauer polynomials, *q*-calculus, Fekete-Szegö problem, Bi-u

## Abstract

This study introduces a new class of bi-univalent functions in the open disk using *q*-Borel distribution series and *q*-Gegenbauer polynomials. It provides estimates for the Taylor coefficients $\left|\mu_{2}\right|$ and $\left|\mu_{3}\right|$ for this family of functions, as well as solutions for the Fekete-Szegö functional problems associated with this subclass. The study presents various innovative findings that result from the unique parameters used in the main results.

## 1 Introduction

In the year 1784, Legendre made the initial discovery of orthogonal polynomials (OP) [1]. When particular model criteria are satisfied, ordinary differential equations are often solved by employing the operator (OP). In addition, the [2] notation plays a significant part in the field of approximation theory.

The polynomials $\Xi_{d}$ and $\Xi_{t}$ of order *d* and *t*, respectively, are said to be orthogonal if

$$\int_{a}^{b}\Xi_{d}(y)\Xi_{t}(y)\varpi (y)dy=0,\;\;\text{for}\;\;d\neq t,$$

where ϖ(y) is a well-defined function in the interval (a,b), and as a result, the integral of all polynomials $\Xi_{n}(y)$ of finite order is well defined.

A type of (OP) is the Gegenbauer polynomial (GP). According to [3], symbolic connections exist between the generating mechanism of (GP) and the integral of the functions $T_{R}$, when conventional algebraic formulations are employed. This is the reason why many valuable inequalities have been discovered in the field of (GP).

The utilization of fractional calculus operators has been widely employed in the explanation and solution of issues in the field of applied sciences, as well as in Geometric Function, as documented in the source [4]. The fractional *q*-calculus is an extension of the standard fractional calculus. For further insights on the topic, it is recommended to consult a published source [5] and current literature, which might include references like [6], [7], [8].

The investigation of q-OP has yielded numerous significant discoveries and methodologies in the field of q-calculus. These encompass the q-analog of the binomial theorem, q-difference equations, and q-special functions. Moreover, the realm of q-OP has been employed to scrutinize q-integrals and q-series, which are indispensable tools in q-calculus. Notably, Quesne [9] recently proposed a reformulation of Jackson's q-exponential as a series of regular exponentials with well-defined coefficients. This breakthrough has significant implications for the theory of q-OP in this specific context and should be duly acknowledged.

The theory of (OP) is a subject that has received significant attention because of its wide range of applications in mathematics and physics. In recent years, there has been an increased utilization of (OP) and their analogues by researchers in the analysis of functions in the complex plane. This is particularly observed in the study of bi-univalent functions (see [10], [11], [12], [13], [14], [15], [16]).

## 2 Preliminaries

Consider the family A consisting of functions Φ of the form

(2.1) $$\Phi (\nu )=\nu +\sum_{n=2}^{\infty }\mu_{n}\nu^{n},\;\;(\nu \in \Omega ),$$

where *ν* belongs to the complex unit disk Ω={ν∈C:|ν|<1}, and Φ is analytic in Ω. Additionally, *f* must satisfy the normalization condition $\Phi^{\prime }(0)=0$ and Φ(0)=1. Furthermore, we denote by S a subclass of A that includes functions of Equation (2.1), which are also univalent in Ω.

The implementation of differential subordination of analytical functions has the potential to offer considerable benefits to the domain of (GFT). Miller and Mocanu [17] introduced the initial differential subordination problem, which has since been further investigated in [18]. In their book [19], Miller and Mocanu provide a comprehensive overview of the developments in this field, including the corresponding publication dates.

It is widely accepted that for every function Φ∈S, there exists an inverse $\Phi^{-1}$ defined by:

$$\Phi^{-1}(\Phi (\nu ))=\nu \;\;\;\text{and}\;\;\;\Phi (\Phi^{-1}(\varrho ))=\varrho ,\;\left(\nu \in \Omega ,\;\;\;\left|\varrho \right|<r_{0}(f);\;r_{0}(\Phi )\geq \frac{1}{4}\right)$$

where

$$\Phi^{-1}(\varrho )=\varrho \left(1-\mu_{2}\varrho +(2\mu_{2}^{2}-\mu_{3})\varrho^{2}-(5\mu_{2}^{3}-5\mu_{2}\mu_{3}+\mu_{4})\varrho^{3}+\cdots \;\right).$$

A function is considered bi-univalent (bi-u) in Ω if both Φ(ν) and $\Phi^{-1}(\nu )$ are univalent within Ω.

The class of (bi-u) functions in Ω, as defined by (2.1), is denoted by Σ. Functions belonging to the class Σ include:

$$\frac{\nu }{1-\nu }\;\;\;\;\text{and}\;\;\;\;\frac{1}{2}\left(\log(1+\nu )-\log(1-\nu )\right).$$

The fundamental probability distributions, such as Poisson, Pascal, Logarithmic, and Binomial, have been investigated to some extent in the GFT from a theoretical perspective (see [20], [21], [22], [23], [24], [25], [26], [27], [28]). More recently, several anthers used the Borel distribution to introduce subsets of analytic and (bi-u) functions, (see for example, [29], [30], [31], [32], [33]).

If a discrete random variable *x* assumes values 1,2,3,… with corresponding probabilities, it is said to follow a Borel distribution

$$\frac{e_{q}^{-\psi }}{{\left[1\right]}_{q}!},\frac{2\psi e_{q}^{-2\psi }}{{\left[2\right]}_{q}!},\frac{9\psi^{2}e_{q}^{-3\psi }}{{\left[3\right]}_{q}!},\frac{64\psi^{3}e_{q}^{-4\psi }}{{\left[4\right]}_{q}!},\ldots$$

respectively, where *ψ* is called the parameter.

Here, we introduce a power series that represents the probabilities linked with the *q*-Borel distribution through its coefficients.

$$M_{q}(\psi ,\nu )=\nu +\sum_{n=2}^{\infty }\frac{{\left(\psi (n-1)\right)}^{n-2}e_{q}^{-\psi (n-1)}}{{\left[n-1\right]}_{q}!}\nu^{n},\;\nu \in \Omega .$$

Now, we observe that the radius of convergence of the aforementioned power series is infinite, as can be deduced from the ratio test, given 0≤ψ≤1.

Moreover, we define the series

$$N_{q}(\psi ,\nu )=2\nu -M_{q}(\psi ,\nu )=\nu -\sum_{n=2}^{\infty }\frac{{\left(\psi (n-1)\right)}^{n-2}e_{q}^{-\psi (n-1)}}{{\left[n-1\right]}_{q}!}\nu^{n},\;\nu \in \Omega .$$

Next, we examine the convolution or Hadamard product, which defines a linear operator $B_{\psi }(\nu ):A⟶A$ on the function space A

(2.2) $$B_{\psi }\Phi (\nu )=N_{q}(\psi ,\nu )⁎\Phi (\nu )=\nu +\sum_{n=2}^{\infty }\frac{{\left(\psi (n-1)\right)}^{n-2}e_{q}^{-\psi (n-1)}}{{\left[n-1\right]}_{q}!}\mu_{n}\nu^{n},\;\nu \in \Omega ,$$

Askey and Ismail [34] developed a class of polynomials that can be regarded of as *q*-analogues of the (GP) in 1983. Furthermore, the recurrence relations provided below can be utilized to interpret a specific set of polynomials, discovered by Chakrabarti et al. in 2006 [35], as *q*-analogues of the (GP):

(2.3) $$\begin{array}{cc} C_{0}^{\left(ℸ\right)}(x;q) & =1\\ C_{1}^{\left(ℸ\right)}(x;q) & =2{\left[ℸ\right]}_{q}x\\ C_{2}^{\left(ℸ\right)}(x;q) & =2\left({\left[ℸ\right]}_{q^{2}}+{\left[ℸ\right]}_{q}^{2}\right)x^{2}-{\left[ℸ\right]}_{q^{2}} \end{array}$$

Amourah et al. [36] and Alsoboh et al. [37] recently introduced subclasses of analytical and bi-univalent functions that use *q*-(OP). In addition, Fekete-Szegö inequalities and constraints for the coefficients $\left|\mu_{2}\right|$ and $\left|\mu_{3}\right|$ are determined for functions belonging to these subclasses.

Bi-univalent functions related to (OP) have recently been the subject of research by various writers, few to mention ([38], [39], [40], [41], [42], [43], [44], [45], [46], [47], [48]).

The primary purpose of this paper is to initiate an investigation into the properties of bi-univalent functions associated to *q*-(GP).

## 3 Definition and examples

The *q*−(GP) is subordinate to some new bi-univalent function subclasses that we introduce in this section.

Definition 3.1 Let Ϝ≥0 and $x\in \left(\frac{1}{2},1\right]$. A function f∈Σ defined by equation (2.1) is considered to be a member of the class $W_{\Sigma }(\psi ,Ϝ;x,ℸ;q)$ if it fulfills the following conditions of subordination.

$$Ϝ\partial_{q}B_{\psi }\Phi (\nu )+\nu^{-1}B_{\psi }\Phi (\nu )-Ϝ\nu^{-1}B_{\psi }\Phi (\nu )≺G_{q}^{\left(ℸ\right)}(x,\nu ),$$

and

$$Ϝ\partial_{q}B_{\psi }g(\varrho )+\varrho^{-1}B_{\psi }g(\varrho )-Ϝ\varrho^{-1}B_{\psi }g(\varrho )≺G_{q}^{\left(ℸ\right)}(x,\varrho ),$$

where $g=\Phi^{-1}$ is defined by (2.2) and $G_{q}^{\left(ℸ\right)}$ is the generating function of *q*-analogues of the (GP) given by (2.3).

Example 3.2 Let $q\to 1^{-}$ and $x\in \left(\frac{1}{2},1\right]$. A function Φ∈Σ given by (2.1) is said to be in the class $W_{\Sigma }(\psi ,Ϝ;x,ℸ;q)$ if it satisfies the following subordination conditions

$$Ϝ{\left(B_{\psi }\Phi (\nu )\right)}^{\prime }+\nu^{-1}B_{\psi }\Phi (\nu )-Ϝ\nu^{-1}B_{\psi }\Phi (\nu )≺G_{q}^{\left(ℸ\right)}(x,\nu ),$$

and

$$Ϝ{\left(B_{\psi }g(\varrho )\right)}^{\prime }+\varrho^{-1}B_{\psi }g(\varrho )-Ϝ\varrho^{-1}B_{\psi }g(\varrho )≺G_{q}^{\left(ℸ\right)}(x,\varrho ).$$

Example 3.3 If Ϝ=0 and *x* belongs to the interval $\left(\frac{1}{2},1\right]$, a function Φ in the class Σ given by (2.1) is considered to be in the class $W_{\Sigma }(\psi ,0;x,ℸ;q)$ if it satisfies the following subordination conditions:

$$\nu^{-1}B_{\psi }\Phi (\nu )≺G_{q}^{\left(ℸ\right)}(x,\nu )\;\;\;\text{and}\;\;\;\varrho^{-1}B_{\psi }g(\varrho )≺G_{q}^{\left(ℸ\right)}(x,\varrho ).$$

Example 3.4 If Ϝ=1 and *x* belongs to the interval $\left(\frac{1}{2},1\right]$, a function Φ in the class Σ given by (2.1) is considered to be in the class $W_{\Sigma }(\psi ,1;x,ℸ;q)$ if it satisfies the following subordination conditions:

$$\partial_{q}B_{\psi }\Phi (\nu )≺G_{q}^{\left(ℸ\right)}(x,\nu )\;\;\;\text{and}\;\;\;\partial_{q}B_{\psi }g(\varrho )≺G_{q}^{\left(ℸ\right)}(x,\varrho ).$$

## 4 Coefficient bounds of the class $W_{\Sigma }(\psi ,Ϝ;x,ℸ;q)$

First, we present the coefficient estimates for the class defined in Definition 1, which are denoted by $W_{\Sigma }(\psi ,Ϝ;x,ℸ;q)$.

Theorem 4.1 *Let given by*(2.1)*belong to the class*$W_{\Sigma }(\psi ,Ϝ;x,ℸ;q)$*, then*

$$|\mu_{2}|\leq \frac{2xe_{q}^{\psi }|{\left[ℸ\right]}_{q}|\sqrt{\frac{2{\left[ℸ\right]}_{q}x}{{\left[2\right]}_{q}!}}}{\sqrt{2(4(1+\left({\left[3\right]}_{q}-1\right)Ϝ)\psi {\left[ℸ\right]}_{q}^{2}-{\left(1+qϜ\right)}^{2}{\left[2\right]}_{q}!\left({\left[ℸ\right]}_{q^{2}}+{\left[ℸ\right]}_{q}^{2}\right))x^{2}+{\left(1+qϜ\right)}^{2}{\left[2\right]}_{q}!{\left[ℸ\right]}_{q^{2}}}}.|\mu_{3}|\leq {\left(\frac{2{\left[ℸ\right]}_{q}xe_{q}^{\psi }}{1+({\left[2\right]}_{q}-1)Ϝ}\right)}^{2}+\frac{{\left[2\right]}_{q}!e_{q}^{2\psi }{\left[ℸ\right]}_{q}x}{(1+Ϝ({\left[3\right]}_{q}-1))\psi }.$$

*Proof.* Let $f\in W_{\Sigma }(\psi ,Ϝ;x,ℸ;q)$. From Definition 3.1, for some analytical ϖ,υ such that ϖ(0)=υ(0)=0 and |ϖ(ν)|<1, |υ(ϱ)|<1 for all ν,ϱ∈Ω, then we can write

(4.1) $$Ϝ\partial_{q}B_{\psi }\Phi (\nu )+\nu^{-1}B_{\psi }\Phi (\nu )-Ϝ\nu^{-1}B_{\psi }\Phi (\nu )≺G_{q}^{\left(ℸ\right)}(x,\varpi (\nu )),$$

(4.2) $$Ϝ\partial_{q}B_{\psi }g(\varrho )+\varrho^{-1}B_{\psi }g(\varrho )-Ϝ\varrho^{-1}B_{\psi }g(\varrho )≺G_{q}^{\left(ℸ\right)}(x,\upsilon (\varrho )).$$

We determine the following from the equality between (4.1) and (4.2)

(4.3) $$\begin{array}{cc} & Ϝ\partial_{q}B_{\psi }\Phi (\nu )+\nu^{-1}B_{\psi }\Phi (\nu )-Ϝ\nu^{-1}B_{\psi }\Phi (\nu )\\ & =1+C_{1}^{\left(ℸ\right)}(x;q)c_{1}\nu +\left[C_{1}^{\left(ℸ\right)}(x;q)c_{2}+C_{2}^{\left(ℸ\right)}(x;q)c_{1}^{2}\right]\nu^{2}+\cdots , \end{array}$$

and

(4.4) $$\begin{array}{cc} & Ϝ\partial_{q}B_{\psi }g(\varrho )+\varrho^{-1}B_{\psi }g(\varrho )-Ϝ\varrho^{-1}B_{\psi }g(\varrho )\\ & =1+C_{1}^{\left(ℸ\right)}(x;q)d_{1}\varrho +\left[C_{1}^{\left(ℸ\right)}(x;q)d_{2}+C_{2}^{\left(ℸ\right)}(x;q)d_{1}^{2}\right])\varrho^{2}+\cdots . \end{array}$$

It is widely assumed that if

$$\left|\varpi (\nu )\right|=\left|c_{1}\nu +c_{2}\nu^{2}+c_{3}\nu^{3}+\cdots \right|<1,\;\;(\nu \in \Omega )$$

and

$$\left|v(\varrho )\right|=\left|d_{1}\varrho +d_{2}\varrho^{2}+d_{3}\varrho^{3}+\cdots \right|<1,\;\;(\varrho \in \Omega ),$$

then,

(4.5) $$|c_{k}|\leq 1\;\text{and}\;|d_{k}|\leq 1\;\text{for all}\;k\in N.$$

The comparable coefficients in (4.3) and (4.4) are compared in this manner, and the result is

(4.6) $$\frac{1+({\left[2\right]}_{q}-1)Ϝ}{e_{q}^{\psi }}\mu_{2}=C_{1}^{\left(ℸ\right)}(x;q)c_{1},$$

(4.7) $$\frac{2(1+Ϝ({\left[3\right]}_{q}-1))\psi }{{\left[2\right]}_{q}!e_{q}^{2\psi }}\mu_{3}=C_{1}^{\left(ℸ\right)}(x;q)c_{2}+C_{2}^{\left(ℸ\right)}(x;q)c_{1}^{2},$$

(4.8) $$-\frac{1+({\left[2\right]}_{q}-1)Ϝ}{e_{q}^{\psi }}\mu_{2}=C_{1}^{\left(ℸ\right)}(x;q)d_{1},$$

and

(4.9) $$\frac{2(1+Ϝ({\left[3\right]}_{q}-1))\psi }{{\left[2\right]}_{q}!e_{q}^{2\psi }}\left(2\mu_{2}^{2}-\mu_{3}\right)=C_{1}^{\left(ℸ\right)}(x;q)d_{2}+C_{2}^{\left(ℸ\right)}(x;q)d_{1}^{2}.$$

It follows from (4.6) and (4.8) that

(4.10) $$c_{1}=-d_{1}$$

and

(4.11) $$2{\left(\frac{1+({\left[2\right]}_{q}-1)Ϝ}{e_{q}^{\psi }}\right)}^{2}\mu_{2}^{2}={\left[C_{1}^{\left(ℸ\right)}(x;q)\right]}^{2}\left(c_{1}^{2}+d_{1}^{2}\right).$$

If we add (4.7) and (4.9), we get

(4.12) $$\frac{4(1+\left({\left[3\right]}_{q}-1\right)Ϝ)\psi }{{\left[2\right]}_{q}!e_{q}^{2\psi }}\mu_{2}^{2}=C_{1}^{\left(ℸ\right)}(x;q)(c_{2}+d_{2})+C_{2}^{\left(ℸ\right)}(x;q)(c_{1}^{2}+d_{1}^{2})$$

By plugging in the expression for $\left(c^{2}1+d^{2}1\right)$ from equation (4.11) into the right-hand side of equation (4.12), we can infer that

(4.13) $$\left(\frac{2(1+\left({\left[3\right]}_{q}-1\right)Ϝ)\psi }{{\left[2\right]}_{q}!}-{\left(1+({\left[2\right]}_{q}-1)Ϝ\right)}^{2}\frac{C_{2}^{\left(ℸ\right)}(x;q)}{{\left[C_{1}^{\left(ℸ\right)}(x;q)\right]}^{2}}\right)\frac{2}{e_{q}^{2\psi }}\mu_{2}^{2}=C_{1}^{\left(ℸ\right)}(x;q)(c_{2}+d_{2})$$

Moreover, using (2.3) and (4.13), we find that

(4.14) $$\begin{array}{cc} \left|\mu_{2}\right| & \leq \frac{2e_{q}^{\psi }|{\left[ℸ\right]}_{q}|x\sqrt{{\left[2\right]}_{q}!{\left[ℸ\right]}_{q}x}}{\sqrt{2\left(4(1+\left({\left[3\right]}_{q}-1\right)Ϝ)\psi {\left[ℸ\right]}_{q}^{2}-{\left[2\right]}_{q}!{\left(1+qϜ\right)}^{2}\left({\left[ℸ\right]}_{q^{2}}+{\left[ℸ\right]}_{q}^{2}\right)\right)x^{2}+{\left[2\right]}_{q}!{\left(1+qϜ\right)}^{2}{\left[ℸ\right]}_{q^{2}}}}. \end{array}$$

Also, if we subtract (4.9) from (4.7), we get

(4.15) $$\frac{4(1+Ϝ({\left[3\right]}_{q}-1))\psi }{{\left[2\right]}_{q}!e_{q}^{2\psi }}\left(\mu_{3}-\mu_{2}^{2}\right)=C_{1}^{\left(ℸ\right)}(x;q)(c_{2}-d_{2})+C_{2}^{\left(ℸ\right)}(x;q)(c_{1}^{2}-d_{1}^{2}).$$

Then, in view of (4.11) and (4.15) becomes

$$|\mu_{3}|\leq {\left(\frac{2{\left[ℸ\right]}_{q}xe_{q}^{\psi }}{1+({\left[2\right]}_{q}-1)Ϝ}\right)}^{2}+\frac{{\left[2\right]}_{q}!e_{q}^{2\psi }{\left[ℸ\right]}_{q}x}{(1+Ϝ({\left[3\right]}_{q}-1))\psi }.$$

In light of the outcome established by Zaprawa [49], we investigate the ensuing Fekete-Szegö inequality that concerns functions belonging to the class $W_{\Sigma }(\psi ,Ϝ;x,ℸ;q)$.

Theorem 4.2 *Let given by* (2.1) *belong to the class* $W_{\Sigma }(\psi ,Ϝ;x,ℸ;q)$ *, then*

$$|\mu_{3}-\sigma \mu_{2}^{2}|\leq \left\{ \begin{array}{cc} \frac{{\left[2\right]}_{q}!e_{q}^{2\psi }\left|{ℸ}_{q}\right|x}{(1+Ϝ({\left[3\right]}_{q}-1))\psi }, & \;\left|\sigma -1\right|\leq \nu ,\\ \left|\frac{8x^{3}{\left[ℸ\right]}_{q}^{3}e_{q}^{2\psi }{\left[2\right]}_{q}!(1-\sigma )}{8{\left[ℸ\right]}_{q}^{2}x^{2}(1+\left({\left[3\right]}_{q}-1\right)Ϝ)\psi -{\left[2\right]}_{q}!{\left(1+({\left[2\right]}_{q}-1)Ϝ\right)}^{2}(2\left({\left[ℸ\right]}_{q^{2}}+{\left[ℸ\right]}_{q}^{2}\right)x^{2}-{\left[ℸ\right]}_{q^{2}})}\right|, & \;\left|\sigma -1\right|\geq \nu , \end{array}\right.$$

*where*

$$\nu =\left|1-\frac{{\left[2\right]}_{q}!{\left(1+({\left[2\right]}_{q}-1)Ϝ\right)}^{2}C_{2}^{\left(ℸ\right)}(x;q)}{2(1+Ϝ({\left[3\right]}_{q}-1))\psi {\left[C_{1}^{\left(ℸ\right)}(x;q)\right]}^{2}}\right|.$$

*Proof.* From (4.13) and (4.15)

$$\begin{array}{cc} & \mu_{3}-\sigma \mu_{2}^{2}=\\ & \frac{(1-\sigma )e_{q}^{2\psi }{\left[2\right]}_{q}!{\left[C_{1}^{\left(ℸ\right)}(x;q)\right]}^{3}}{2\left(2(1+\left({\left[3\right]}_{q}-1\right)Ϝ)\psi {\left[C_{1}^{\left(ℸ\right)}(x;q)\right]}^{2}-{\left[2\right]}_{q}!{\left(1+({\left[2\right]}_{q}-1)Ϝ\right)}^{2}C_{2}^{\left(ℸ\right)}(x;q)\right)}(c_{2}+d_{2})\\ & +\frac{{\left[2\right]}_{q}!e_{q}^{2\psi }C_{1}^{\left(ℸ\right)}(x;q)}{4(1+Ϝ({\left[3\right]}_{q}-1))\psi }(c_{2}-d_{2})\\ & =C_{1}^{\left(ℸ\right)}(x;q)\left(h(\sigma )+\frac{{\left[2\right]}_{q}!e_{q}^{2\psi }}{4(1+Ϝ({\left[3\right]}_{q}-1))\psi }\right)+C_{1}^{\left(ℸ\right)}(x;q)\left(h(\sigma )-\frac{{\left[2\right]}_{q}!e_{q}^{2\psi }}{4(1+Ϝ({\left[3\right]}_{q}-1))\psi }\right) \end{array}$$

where

$$h(\sigma )=\frac{(1-\sigma )e_{q}^{2\psi }{\left[2\right]}_{q}!{\left[C_{1}^{\left(ℸ\right)}(x;q)\right]}^{2}}{2\left(2(1+\left({\left[3\right]}_{q}-1\right)Ϝ)\psi {\left[C_{1}^{\left(ℸ\right)}(x;q)\right]}^{2}-{\left[2\right]}_{q}!{\left(1+({\left[2\right]}_{q}-1)Ϝ\right)}^{2}C_{2}^{\left(ℸ\right)}(x;q)\right)}.$$

Then, in view of (2.3), we conclude that

$$|\mu_{3}-\sigma \mu_{2}^{2}|\leq \left\{ \begin{array}{cc} \frac{{\left[2\right]}_{q}!e_{q}^{2\psi }\left|C_{1}^{\left(ℸ\right)}(x;q)\right|}{2(1+Ϝ({\left[3\right]}_{q}-1))\psi }, & \;\left|h(\sigma )\right|\leq \frac{{\left[2\right]}_{q}!e_{q}^{2\psi }}{4(1+Ϝ({\left[3\right]}_{q}-1))\psi },\\ 2\left|C_{1}^{\left(ℸ\right)}(x;q)\right|\left|h(\sigma )\right|, & \;\;\left|h(\sigma )\right|\geq \frac{{\left[2\right]}_{q}!e_{q}^{2\psi }}{4(1+Ϝ({\left[3\right]}_{q}-1))\psi }. \end{array}\right.$$

## 5 Corollaries

Corollaries derived from Theorems 1 and 2 can be roughly associated with Example 3.2, Example 3.3, Example 3.4.

Corollary 5.1 *Suppose f defined by equation*(2.1)*and belonging to the class* Σ*, is also a member of the class* $W_{\Sigma }(\psi ,Ϝ;x,ℸ;1)$*. Then, we can conclude that*

$$|\mu_{2}|\leq \frac{2xe^{\psi }\;|ℸ|\sqrt{ℸx}}{\sqrt{2(4(1+2Ϝ)\psi {ℸ}^{2}-2{\left(1+Ϝ\right)}^{2}ℸ\left(1+ℸ\right))x^{2}+2ℸ{\left(1+Ϝ\right)}^{2}}}.|\mu_{3}|\leq {\left(\frac{2ℸxe^{\psi }}{1+Ϝ}\right)}^{2}+\frac{2e^{2\psi }ℸx}{(1+2Ϝ)\psi },$$

*and*

$$|\mu_{3}-\sigma \mu_{2}^{2}|\leq \left\{ \begin{array}{cc} \frac{2e^{2\psi }\left|{ℸ}_{q}\right|x}{(1+2Ϝ)\psi }, & \;\left|\sigma -1\right|\leq \left|1-\frac{2{\left(1+Ϝ\;\right)}^{2}C_{2}^{\left(ℸ\right)}(x;1)}{2(1+2Ϝ)\psi {\left[C_{1}^{\left(ℸ\right)}(x;1)\right]}^{2}}\right|,\\ \left|\frac{16x^{3}{ℸ}^{3}e^{2\psi }(1-\sigma )}{8{ℸ}^{2}x^{2}(1+2Ϝ)\psi -2{\left(1+Ϝ\right)}^{2}(2ℸ\left(1+ℸ\right)x^{2}-ℸ)}\right|, & \;\left|\sigma -1\right|\geq \left|1-\frac{2{\left(1+Ϝ\;\right)}^{2}C_{2}^{\left(ℸ\right)}(x;1)}{2(1+2Ϝ)\psi {\left[C_{1}^{\left(ℸ\right)}(x;1)\right]}^{2}}\right|. \end{array}\right.$$

Corollary 5.2 *Suppose f defined by equation*(2.1)*and belonging to the class* Σ*, is also a member of the class* $W_{\Sigma }(\psi ,1;x,ℸ;q)$*. Then, we can conclude that*

(5.1) $$|\mu_{2}|\leq \frac{2xe_{q}^{\psi }|{\left[ℸ\right]}_{q}|\sqrt{\frac{2{\left[ℸ\right]}_{q}x}{{\left[2\right]}_{q}!}}}{\sqrt{2(4(1+\left({\left[3\right]}_{q}-1\right))\psi {\left[ℸ\right]}_{q}^{2}-{\left(1+q\right)}^{2}{\left[2\right]}_{q}!\left({\left[ℸ\right]}_{q^{2}}+{\left[ℸ\right]}_{q}^{2}\right))x^{2}+{\left(1+q\right)}^{2}{\left[2\right]}_{q}!{\left[ℸ\right]}_{q^{2}}}}.|\mu_{3}|\leq {\left(\frac{2{\left[ℸ\right]}_{q}xe_{q}^{\psi }}{1+({\left[2\right]}_{q}-1)}\right)}^{2}+\frac{{\left[2\right]}_{q}!e_{q}^{2\psi }{\left[ℸ\right]}_{q}x}{(1+Ϝ({\left[3\right]}_{q}-1))\psi },$$

*and*

$$|\mu_{3}-\sigma \mu_{2}^{2}|\leq \left\{ \begin{array}{cc} \frac{{\left[2\right]}_{q}!e_{q}^{2\psi }\left|{ℸ}_{q}\right|x}{(1+({\left[3\right]}_{q}-1))\psi }, & \;\left|\sigma -1\right|\leq \nu ,\\ \left|\frac{8x^{3}{\left[ℸ\right]}_{q}^{3}e_{q}^{2\psi }{\left[2\right]}_{q}!(1-\sigma )}{8{\left[ℸ\right]}_{q}^{2}x^{2}(1+\left({\left[3\right]}_{q}-1\right))\psi -{\left[2\right]}_{q}!{\left(1+({\left[2\right]}_{q}-1)\right)}^{2}(2\left({\left[ℸ\right]}_{q^{2}}+{\left[ℸ\right]}_{q}^{2}\right)x^{2}-{\left[ℸ\right]}_{q^{2}})}\right|, & \;\left|\sigma -1\right|\geq \nu , \end{array}\right.$$

*where*

$$\nu =\left|1-\frac{{\left[2\right]}_{q}!{\left(1+({\left[2\right]}_{q}-1)\right)}^{2}C_{2}^{\left(ℸ\right)}(x;q)}{2(1+Ϝ({\left[3\right]}_{q}-1))\psi {\left[C_{1}^{\left(ℸ\right)}(x;q)\right]}^{2}}\right|.$$

Corollary 5.3 *Suppose f defined by equation*(2.1)*and belonging to the class* Σ*, is also a member of the class* $W_{\Sigma }(\psi ,0;x,ℸ;q)$*. Then, we can conclude that*

(5.2) $$|\mu_{2}|\leq \frac{2xe_{q}^{\psi }|{\left[ℸ\right]}_{q}|\sqrt{\frac{2{\left[ℸ\right]}_{q}x}{{\left[2\right]}_{q}!}}}{\sqrt{2(4\psi {\left[ℸ\right]}_{q}^{2}-{\left[2\right]}_{q}!\left({\left[ℸ\right]}_{q^{2}}+{\left[ℸ\right]}_{q}^{2}\right))x^{2}+{\left[2\right]}_{q}!{\left[ℸ\right]}_{q^{2}}}}.|\mu_{3}|\leq {\left(2{\left[ℸ\right]}_{q}xe_{q}^{\psi }\right)}^{2}+{\left[2\right]}_{q}!e_{q}^{2\psi }{\left[ℸ\right]}_{q}x\psi ,$$

*and*

$$|\mu_{3}-\sigma \mu_{2}^{2}|\leq \left\{ \begin{array}{cc} {\left[2\right]}_{q}!e_{q}^{2\psi }\left|{ℸ}_{q}\right|x)\psi , & \;\left|\sigma -1\right|\leq \left|1-\frac{{\left[2\right]}_{q}!C_{2}^{\left(ℸ\right)}(x;q)}{2\psi {\left[C_{1}^{\left(ℸ\right)}(x;q)\right]}^{2}}\right|,\\ \left|\frac{8x^{3}{\left[ℸ\right]}_{q}^{3}e_{q}^{2\psi }{\left[2\right]}_{q}!(1-\sigma )}{8{\left[ℸ\right]}_{q}^{2}x^{2}\psi )-{\left[2\right]}_{q}!(2\left({\left[ℸ\right]}_{q^{2}}+{\left[ℸ\right]}_{q}^{2}\right)x^{2}-{\left[ℸ\right]}_{q^{2}})}\right|, & \;\left|\sigma -1\right|\geq \left|1-\frac{{\left[2\right]}_{q}!C_{2}^{\left(ℸ\right)}(x;q)}{2\psi {\left[C_{1}^{\left(ℸ\right)}(x;q)\right]}^{2}}\right|. \end{array}\right.$$

**Conclusion:** We have investigated the coefficient problems associated with three novel subclasses of the bi-univalent function class within the domain of the open unit disk $W_{\Sigma }(\psi ,Ϝ;x,ℸ;q)$, $W_{\Sigma }(\psi ,Ϝ;x,ℸ;1)$, $W_{\Sigma }(\psi ,0;x,ℸ;q)$, and $W_{\Sigma }(\psi ,1;x,ℸ;q)$, as described in Definition 3.1. For each of these subclasses, we have estimated the Taylor-Maclaurin coefficients $\left|\mu_{2}\right|$ and $\left|\mu_{3}\right|$, as well as provided estimates for the Fekete-Szegö functional problems. We also explored additional results that were discovered through specialization of the parameters used in our primary results. In the future, it may be worthwhile to investigate the Hankel determinants of these classes. We anticipate that the *q*-defferintegral operator will have practical applications in various scientific domains, including technology and mathematics.

## CRediT authorship contribution statement

**T. Al-Hawary:** Conceptualization. **A. Alsoboh:** Methodology, Investigation, Formal analysis, Conceptualization. **A. Amourah:** Software, Resources, Methodology, Conceptualization. **O. Ogilat:** Investigation, Data curation. **I. Harny:** Visualization, Validation, Conceptualization. **M. Darus:** Visualization, Validation, Supervision.

## Declaration of Competing Interest

The authors declare that they have no known competing financial interests or personal relationships that could have appeared to influence the work reported in this paper.

## Data Availability

Data included in article/supp. material/referenced in article.
